# Egg nutritional modulation with amino acids improved performance in zebrafish larvae

**DOI:** 10.1371/journal.pone.0248356

**Published:** 2021-04-09

**Authors:** Carmen Navarro-Guillén, Gabriella do Vale Pereira, André Lopes, Rita Colen, Sofia Engrola

**Affiliations:** 1 Aquaculture Research Group, Centro de Ciências do Mar do Algarve (CCMAR), Faro, Portugal; 2 SPAROS Lda., Olhão, Portugal; Kafrelsheikh University, EGYPT

## Abstract

New and more efficient methods to sustainably intensify Aquaculture production are essential to attain the seafood demand for direct human consumption in the near future. Nutrition has been identified as one strategy of early exposure that might affect animal early development and later phenotype. This strategy may have positive consequences in the modulation of fish digestive physiology, which will correlate with higher performance outputs. Thus, improving fish digestive efficiency will lead to higher productivity and lower biogenic emission from aquaculture facilities, minimising the impact on the environment while increasing the biological efficiency. An innovative *in ovo* nutritional modulation technique based on low-frequency ultrasounds was used to enhance the transport of amino acids across the embryo membranes. An early stimulus with either arginine or glutamine, both involved in gut maturation, was applied in zebrafish (*Danio rerio*) embryos at 3.5 hours post-fertilization (hpf). At 22 days post-fertilization (dpf), growth performance, digestive enzyme activities and gut microbiota composition were analysed to evaluate the larval nutrition-induced metabolic plasticity and the effects on fish digestive efficiency. Results showed that fish survival was not affected either by the sonophoresis technique or amino acid supplementation. Final dry weight at 22 dpf was statistically higher in larvae from glutamine treatment when compared to the control even with lower trypsin activity, suggesting a higher nutrient digestion capacity, due to a slightly modulation of gut microbiota. Higher arginine supplementation levels should be tested as strategy to enhance growth at later developmental stages. In conclusion, this study demonstrated the efficiency of sonophoresis technique for *in ovo* nutritional modulation and suggests that *in ovo* glutamine supplementation might promote growth at later developmental stage through a positive microbiota modulation.

## Introduction

Early life environmental interventions are a promising way to modulate regulatory mechanisms, with the potential for improving growth rate, biological efficacy, feed utilization efficiency and robustness. Early programming is defined as an event at early stage that influences in the long-term the animal, as a consequence of adaptive changes at the cellular, molecular and biochemical levels [1].

Nutritional research has demonstrated that dietary amino acids (AAs) in general, and glutamine and arginine in particular, play fundamental roles in the overall digestive capacity [2]. Arginine an indispensable amino acid, plays a fundamental role in intestinal maturation. Particular consideration is due to arginine’s unique role over polyamines biosynthesis, being a direct precursor of putrescine, which in turn is precursor for spermidine and spermine biosynthesis. Numerous studies have shown that polyamines have a significant effect on the growth of the intestinal mucosa of a variety of research animals including fish [3,4], as well as alleviates gut mucosa injury [5,6], increases villus height and decreases crypt depth [7]. Similarly, glutamine is an abundant dispensable amino acid in the plasma of animals. Dietary glutamine supplementation in juvenile Jian carp (*Cyprinus carpio* var. Jian) improved weight gain, digestive proteases, and lipases activities and both intestinal structure and function [8]. Likewise, dietary glutamine improved feeding efficiency, innate immune responses, and intestinal structure in juvenile red drum (*Sciaenops ocellatus*) [7].

One of the most sensitive periods for early programming in fish is during egg phase [9], however the lack of *in ovo* modulation techniques has been a bottleneck in fish nutritional programming. Nowadays, the modulation of the nutritional reserves in a fish egg is possible through a conventional approach, like broodstock nutrition [10], or through innovative techniques like, microinjection [11], or electroporation [12]. However, the latter two techniques are not feasible at industrial hatchery levels production. All these techniques have advantages and disadvantages. Microinjection technique ensures the release of exogenous material into the egg. However, regarding embryo survival, microinjection induced high mortality in larvae of zebrafish (*Danio rerio*), [13], while in Pacific bluefin tuna (*Thunnus orientalis*) eggs microinjection was only considered enabled if procedures and transfer to sea water were accomplished by the 2-cell stage [14]. By contrast, electroporation presents high survival close to 95% when using a low voltage [12], but it may cause irreversible permeabilization of the vitelline membrane via the electric breakdown mechanism [15]. The most used approach, broodstock nutrition, has proven to work, nevertheless, it is important to take into consideration the metabolism of adults (especially during oocyte maturation) and the process of nutrient incorporation into developing oocytes [10]. The benefit of early programming *via* maternally-derived nutrition is that it can have direct impacts on offspring as soon as at embryo and the endogenous feeding periods [16], and some effects could be maintained in the long-term [17].

Sonophoresis is defined as the use of ultrasonic energy to enhance the topical or transdermal delivery of drugs or compounds [18]. An ultrasound is an acoustic vibration characterized by longitudinal compression waves that propagate at frequencies beyond the human auditory threshold. Thus, ultrasound transmission induces compression and expansion of the medium through which it is passing, leading to associated pressure variations. The first experiment focused on sonophoresis for enhancing drug delivery was done to enhance transcutaneous insulin penetration *in vivo* hairless rats [19]. An array of subsequent studies confirmed that low-frequency (20–500 kHz) ultrasound was indeed highly effective in enhancing the transdermal absorption of a diverse range of molecules. The application of sonophoresis in fish was mainly explored to increase the permeability of external tissues to enhance the uptake of immersion vaccines. In goldfish (*Carassius auratus*), ultrasound during immersion increased both, the amount of silver chloride particles that penetrated into the epidermis, as well as the depth to which they penetrated [20]. Short pulses of ultrasound during DNA vaccination of rainbow trout (*Oncorhynchus mykiss*) against viral haemorrhagic septicaemia virus (VHSH) was the only method resulting in both, humoral antibody responses and survival after VHSV challenge [21]. After using sonophoresis to facilitate the vaccination of grouper (*Epinephalus awoara*) against *Vibrio alginolyticus*, it was concluded that ultrasound-facilitated vaccination provided an effective and practical approach for large scale vaccination [22]. In early stages of rainbow trout larvae development, cavitation level ultrasound was used to enhance the diffusion of calcein for the purpose of mass-marking [23]. This study tested two voltage amplitudes, two calcein concentrations and two duration of treatments. This study demonstrated that higher diffusion of calcein into fish can be achieved when treated with cavitation level low frequency ultrasound. Therefore, these studies suggested that sonophoresis technique has potential to *in vivo* deliver compounds by increasing the permeability of external tissues and consequently to enhance the uptake to animal tissues.

The present study aimed to assess the feasibility of early supplementation of the amino acids, glutamine, and arginine, as promoters of intestinal maturation at mouth opening stage larva. For that, an innovative *in ovo* nutritional technique based on low-frequency ultrasounds (sonophoresis) was used to enhance the transport of the amino acids across the fish embryo membranes. Growth performance, digestive enzyme activities and gut microbiota were analysed to evaluate the larval nutrition-induced metabolic plasticity and the effects of early programming on fish digestive efficiency.

## Materials and methods

This experiment was performed by trained scientists and following the European Directive 2010/63/EU of European Parliament and of the Council of European Union on the protection of animals used for scientific purposes and was approved by the Committee of Ethic and Animal Experimentation of the Centre of Marine Sciences of Algarve (CCMAR-CBMR ORBEA). CCMAR facilities and their staff are certified to house and conduct experiments with live animals (‘group-1’ license by the ‘Direção Geral de Veterinaria’, Ministry of Agriculture, Rural Development and Fisheries of Portugal).

### Zebrafish eggs supplementation through sonophoresis

The supplementation was performed using sonophoresis, a low frequency ultrasounds-based technique. The sonophoresis prototype system is comprised of a signal generator, a signal amplifier, and an ultrasound immersion transducer, which is submerged in the solution containing the eggs and the compound to be incorporated in. The ultrasound transducer has a diameter of 2.5cm and is designed for a centre frequency of 1kHz. The protocol for zebrafish eggs modulation consisted in two pulses each one with 100 sec of duration (5 seconds of interval between pulses), with a frequency of 1x10^5^ Hz, and amplitude between 100 mV. Fertilized eggs were obtained from a breeding population of zebrafish wild-type AB strain (ZFIN ID: ZDB-GENO-960809-7) maintained at the Centre of Marine Sciences (CCMAR, Portugal) for more than 10 generations. Between 3.5- and 4.5-hours post-fertilization (hpf) zebrafish eggs were submitted to three different treatments, in triplicate; Control (CRTL)–no supplementation; arginine supplementation (ARG) and glutamine supplementation (GLN). Amino acids were dissolved in Ringer solution for teleost fish [24] to a final concentration of 0.44 and 0.96 mM for arginine (ARG) and glutamine (GLN), respectively. Concentrations were selected based on preliminary in-house trials and using the free amino acids profile of zebrafish eggs as reference (unplublished data). For each replicate, 120 eggs were placed in a glass 250 ml measuring cylinder. This cylinder had the same diameter as the ultrasound transducer, forcing the ultrasound waves to go down in the solution column. In addition, since freshwater fish eggs sink, the diameter of the measuring cylinder was big enough to allow all the eggs to deposit in a single layer, being equally exposed to the ultrasound waves. After placing the eggs, the ultrasound transducer was immersed in the corresponding solution within the measuring cylinder and the sonophoresis protocol was applied. After sonophoresis, a pool of 50 eggs per replicate (*n* = 3 pools per treatment) were placed in incubation chambers and sampled 1h after sonophoresis for incorporation efficiency. This period is a suitable trade-off between the time necessary for the recovery of embryo membrane permeability while avoiding significant supplementation losses by osmotic regulation. Eggs were washed twice in distilled water before sampling. With the objective of assessing the effect of sonophoresis technique on fish viability, embryos that were not subjected to the sonophoresis technique (NO SONO) were reared in triplicate at the same density and conditions.

### Rearing conditions

After sonophoresis, embryos were incubated in triplicate tanks at an initial density of 70 individuals l^−1^. Larvae were reared under standardized and optimal conditions described for the species until 22 dpf [25], with a water temperature of 28.3 ± 0.5°C and photoperiod of 14L:10D. Larvae were fed *ad libitum* on a commercial inert diet (SPAROS Lda., Portugal) from mouth opening. At 5 dpf, a pool of 6 larvae was collected from each replicate (*n* = 3 pools per treatment) to determinate the larva individual dry weight at the beginning of exogenous feeding. At the end of the experiment (22 dpf) larvae were sampled to assess the effect of *in ovo* amino acid supplementation on growth performance, free amino acids (FAAs) profile, digestive enzyme activities and gut microbiota composition in later developmental stages. At sampling, larvae were euthanized with an overdose of MS222 (Sigma-Aldrich) and rinsed in distilled water. Twelve individual larvae per replicate (*n* = 36 fish per treatment) were sampled for dry weight determination, 3 pooled larvae per replicate (*n* = 3 pools treatment) for FAAs profile, 15 individual larvae per replicate (*n* = 45 fish per treatment) for digestive enzyme activities measurement and 6 pooled digestive systems per replicate (*n* = 3 pools per treatment) were collected for gut microbiota composition analysis. All samples were snap-frozen in liquid nitrogen after collection and kept at -80°C until analysis. For the determination of individual dry weight, larvae were freeze-dried and weighted in a precision scale (±0.001 mg, Sartorius MSA365-000-DH). All zebrafish larvae were counted to determine survival.

### Incorporation efficiency

To evaluate the amino acid incorporation efficiency of sonophoresis technique the FAAs profile of the zebrafish embryos was analysed. FAAs profile of the larvae at the end of the experiment (22 dpf) were also analysed to evaluate the larval metabolic modulation of FAAs along the experimental period. Prior to analysis all samples were freeze-dried. FAAs analysis of zebrafish embryos and larvae was performed after homogenization in 0.1 M HCl on ice, centrifugation at 1500g at 4°C for 15 min and deproteinization of the supernatant by centrifugal ultrafiltration (3 kDa cut-off, 13500g at 4°C for 20 min). Samples were precolumn derivatized with Waters AccQ Fluor Reagent (6-aminoquinolyl-N-hydroxysuccinimidyl carbamate) using the AccQ Tag method (Waters, Milford, MA). Analyses were performed by ultra-high-performance liquid chromatography (UPLC) on a Waters Reversed-Phase Amino Acid Analysis System, using norvaline as an internal standard. Amino acids were identified by retention times of standard mixtures (Waters) and pure standards (Sigma, Madrid, Spain). Instrument control and data acquisition and processing were achieved through Waters Empower software.

### Digestive enzyme activities

Digestive enzyme activities were analysed at the end of the experiment (22 dpf) to study the effects of early nutrition on digestive capacity. To prepare the enzyme extracts, larvae were previously freeze-dried, and every single larva was manually homogenized in 210 μl distilled water. The homogenate was centrifuged for 10 min at 11000g, 4°C to remove the tissue, after, the supernatant extract was used for the analysis of trypsin, chymotrypsin, aminopeptidase N, lipases, and alkaline phosphatase activities. All samples were kept in ice during the process described above to avoid enzymes denaturation and/or damage. Enzyme extracts were kept at −20°C until further analysis.

For trypsin, chymotrypsin, and aminopeptidase N analysis, the fluorogenic substrates Boc-Gln-Ala-Arg-7- methylcoumarin hydrochloride (BOC—SIGMA B4153), N-Succinyl-Ala-Ala-Pro-Phe-7-amido-4-metilcoumarin (SIGMA S9761) and Nα-Benzoyl-L-arginine-7-amido-4-methylcoumarin hydrochloride (SIGMA B7260), respectively, were diluted in dimethyl sulfoxide (DMSO), to a final concentration of 20 μM. For analysis, 190 μl of 50 mM Tris + 10 mM CaCl2 buffer (pH 8.5), 15 μl of the extract homogenate and 5 μl of the flourogenic substrate were added to the microplate. Fluorescence was measured at 355 nm (excitation) and 460 nm (emission).

Lipases activities were assayed using 4-methylumbelliferyl heptanoate (Sigma-Aldrich) and 4-methylumbelliferyl oleate (Sigma-Aldrich). The substrates were dissolved in phosphate buffer pH 7.0 to a final concentration of 0.4 mM, modified method from Rotllant [26], aliquoted and stored at −20°C. 15 μl of the larvae homogenate was added to the microplate and mixed with 250 μl of 0.4 mM substrate for the analysis. Fluorescence was measured at 355 nm (excitation) and 460 nm (emission).

For alkaline phosphatase analysis the substrate 4-methylumbelliferyl phosphate disodium salt (4-MUP, M8168 Sigma) was diluted in borate buffer (pH 8.5) to a final concentration of 1 mM. In the microplate, 100 ul of substrate and 15 ul of extract were mixed. Fluorescence was measured at 360 nm (excitation) and 440nm (emission).

All enzyme activities were expressed as RFU (Relative Fluorescence Units) per mg larvae dry weight.

### Gut microbiota composition

At 22 dpf, zebrafish larvae were euthanized with an overdose of MS222 (Sigma-Aldrich, Madrid, Spain) and rinsed with ethanol to avoid contamination of the intestinal samples with mucosal microbiota. The gastrointestinal tract (GIT) of each laterally placed larva was aseptically excised under a Leica microscope (Leica Microsystems, Wetzlar, Germany). Each sample was a pool of GITs from 6 larvae; one sample was collected from each replicate tank (*n* = 3 pools per treatment). Pool of GITs were immediately transferred to DNA/RNA ShieldTM tubes (Zymo Research, Irvine, CA) and sent for analysis. The samples were processed and analysed with the ZymoBIOMICS^®^ Targeted Sequencing Service for Microbiome Analysis (Zymo Research, Irvine, CA).

The ZymoBIOMICS^®^-96 MagBead DNA Kit (Zymo Research, Irvine, CA) was used to extract DNA using an automated platform. Bacterial 16S ribosomal RNA gene targeted sequencing was performed using the Quick-16S^™^ NGS Library Prep Kit (Zymo Research, Irvine, CA). The bacterial 16S primers amplified the V3-V4 region of the 16S rRNA gene. These primers have been custom-made by Zymo Research to provide the best coverage of the 16S gene while maintaining high sensitivity. The sequencing library was prepared using an innovative library preparation process in which PCR reactions were performed in real-time PCR machines to control cycles and therefore prevent limit PCR chimera formation. The final PCR products were quantified with qPCR fluorescence readings and pooled together based on equal molarity. The final pooled library was cleaned up with the Select-a-Size DNA Clean & Concentrator^™^ (Zymo Research, Irvine, CA), then quantified with TapeStation^®^ (Agilent Technologies, Santa Clara, CA) and Qubit^®^ (Thermo Fisher Scientific, Waltham, WA). The final library was sequenced on Illumina^®^ MiSeq^™^ with a v3 reagent kit (600 cycles). The sequencing was performed with >10% PhiX spike-in.

The ZymoBIOMICS^®^ Microbial Community Standard (Zymo Research, Irvine, CA) was used as a positive control for each DNA extraction. The ZymoBIOMICS® Microbial Community DNA Standard (Zymo Research, Irvine, CA) was used as a positive control for each targeted library preparation. Negative controls (i.e. blank extraction control, blank library preparation control) were included to assess the level of bioburden carried by the wet-lab process.

### Statistical analysis

All data of individual dry weight (mg), FAAs profile, activity of digestive enzymes and gut microbiota composition from zebrafish eggs and larvae are shown as means ± standard deviation (SD). Percentage data (survival) were arcsine square root-transformed prior to analysis. Homogeneity of variance was checked by Levene’s test. Survival between NO SONO and CTRL treatments was compared by means of unpaired two-tailed Student’s *t*-test. To analyse differences in survival, growth performance and digestive enzyme activities between CTRL, ARG and GLN treatments one-way ANOVA with Tukey post hoc test was used with SPSS 19 software (IBM, New York, U.S.A.). The similarities of the samples for FAA profile and digestive enzymes activity levels were represented in Principal Component Analysis using Primer V7 software (Primer-e Ltd., New Zealand). The differences among treatments were considered significant at P ≤ 0.05.

Regarding bioinformatics for microbiota analysis unique amplicon sequences were inferred from raw reads using the Dada2 pipeline [27]. Chimeric sequences were also removed with the Dada2 pipeline. Taxonomy assignment was performed using Uclust from Qiime v.1.9.1 Taxonomy with the Zymo Research Database, a 16S database that is internally designed and curated, as reference. Composition visualization, alpha-diversity, and beta-diversity analyses were performed with Qiime v.1.9.1 [28]. If applicable, taxonomy that have significant abundance among different groups were identified by LEfSe [29] using default settings. PCoA plots were performed with internal scripts. Weighted and unweighted UniFrac dissimilarity matrixes were imported to Primer to evaluate significant differences between groups by permutational multivariate analysis of variance (PERMANOVA) with Permanova+.

## Results

**Fig 1:**
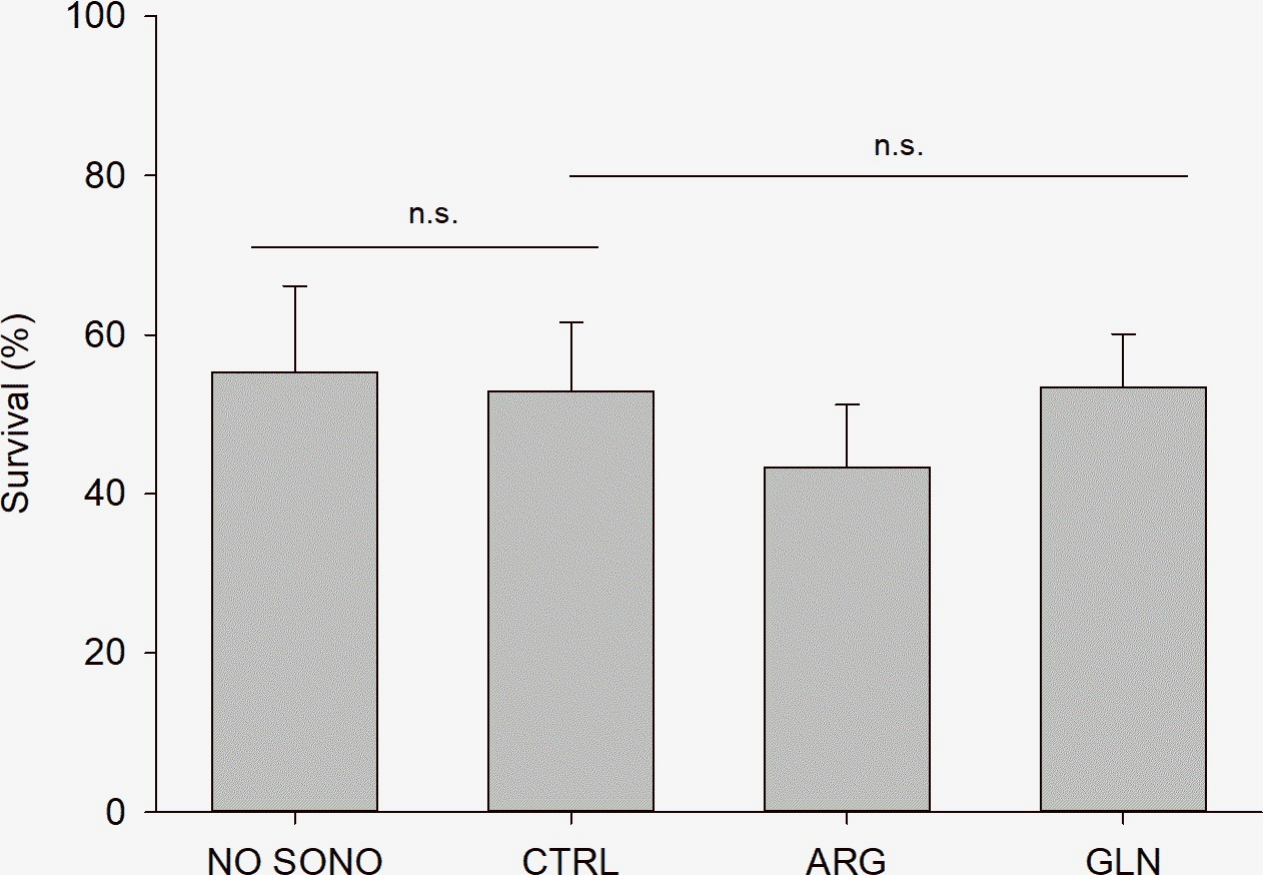
Zebrafish larvae survival at the end of the experiment. Survival (%) of zebrafish larvae (22 dpf) from NO SONO, CTRL, ARG and GLN treatments. Values are presented as mean ± SD. “n.s.” denotes absence of statistical difference among treatments (P > 0.05).

Sonophoresis technique did not show significant negative effect on fish survival when compared to the control group (p = 0.775). Likely, amino acids (ARG and GLN) supplementation did not negatively affect survival (p = 0.293). Overall, survival at the end of the experiment was 51.19±10.38% (Fig 1).

**Fig 2:**
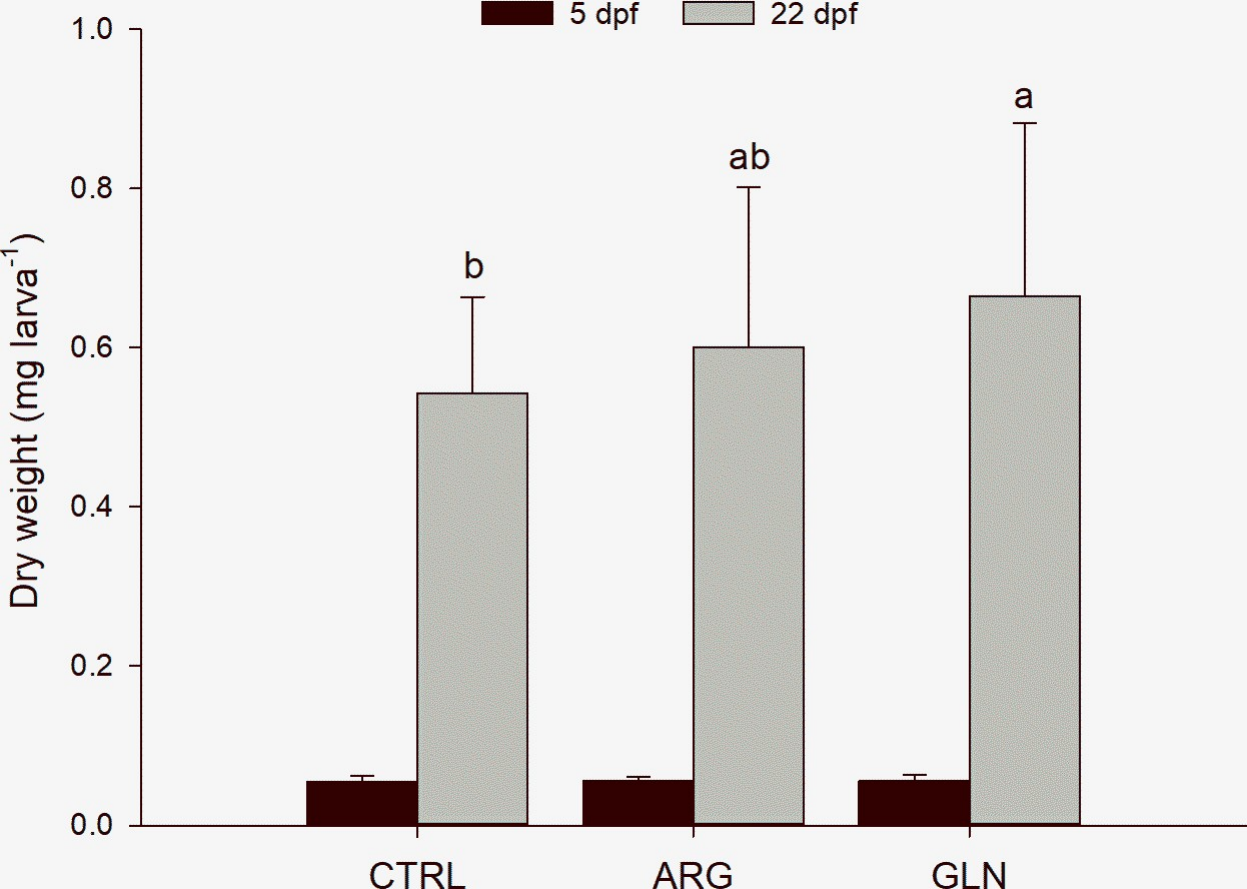
Zebrafish larvae growth performance. Growth performance (individual dry weight [mg]) of zebrafish larvae from CTRL, ARG and GLN treatments at 5 (*n* = 3 pools of 6 larvae per treatment) and 22 dpf (*n* = 36 individual larvae per treatment) (black and grey bars, respectively). Values are presented as mean ± SD. Different letters denote statistical difference among treatments (P < 0.05).

At the beginning of the exogenous feeding, fish dry weight did not differ between treatments, with an average dry weight of 0.055±0.002 mg larva^-1^. By contrast, at the end of the experiment GLN larvae reached significantly higher dry weight than CTRL larvae (p = 0.027). Final dry weight ranged from 0.54±0.12 to 0.66±0.22 mg larva^-1^, CTRL and GLN respectively (Fig 2).

### Incorporation efficiency

Free amino acids profile of zebrafish embryos (1h after sonophoresis) and larvae (22dpf) are shown in Table 1. The FAAs profile confirmed the effectiveness of sonophoresis for arginine and glutamine supplementation into zebrafish eggs, with statistically higher levels of arginine and glutamine recorded in ARG and GLN embryos, respectively (P = 0.000 and 0.000, respectively) (S1A Fig). In addition, the effects of the early supplementation in the larval FAAs profile were maintained until the end of the experiment (22 dpf) (P = 0.000 and 0.000 for ARG and GLN, respectively) (S1B Fig). As expected, amino acid supplementation also affected the pool of total indispensable and dispensable amino acids in both, embryos, and larvae. Arginine supplementation (indispensable amino acid) significantly increased the total IAA pool, while glutamine supplementation (dispensable amino acid) significantly increased the total pool of DAA.

**Table 1:** Zebrafish embryos free amino acids profile.

	Embryos			Larvae		
	CTRL	ARG	GLN	CTRL	ARG	GLN
Arginine	7.68±0.11^b^	15.16±1.01^a^	7.48±0.22^b^	7.87±0.25^b^	10.98±0.46^a^	7.08±0.47^b^
Glutamine	3.35±0.10^b^	3.26±0.12^b^	12.89±0.35^a^	3.23±0.09^b^	2.68±0.19^c^	5.51±0.15^a^
Σ IAA	33.07±0.57^b^	39.04±1.91^a^	31.33±0.90^b^	25.28±0.36^a^	26.98±1.08^a^	22.84±1.04^b^
Σ DAA	44.11±0.36^b^	41.61±1.79^b^	51.69±2.35^a^	38.82±0.38^a^	33.98±1.30^b^	36.37±1.64^ab^
IAA:DAA	0.75±0.01^b^	0.94±0.01^a^	0.61±0.01^c^	0.65±0.01^b^	0.79±0.04^a^	0.63±0.03^b^

Results are shown as mean ± SD. Different letters mean statistical differences for each amino acid between treatments at the same developmental stage (P<0.05). Indispensable (IAA) and dispensable (DAA) amino acids for fish according to Halver [30].

Free amino acids profile (mg AA/g DW) of zebrafish embryos 1h after sonophoresis and 22 dpf larvae from control (CTRL), arginine (ARG) and glutamine (GLN) treatments.

### Digestive enzyme activities

**Fig 3:**
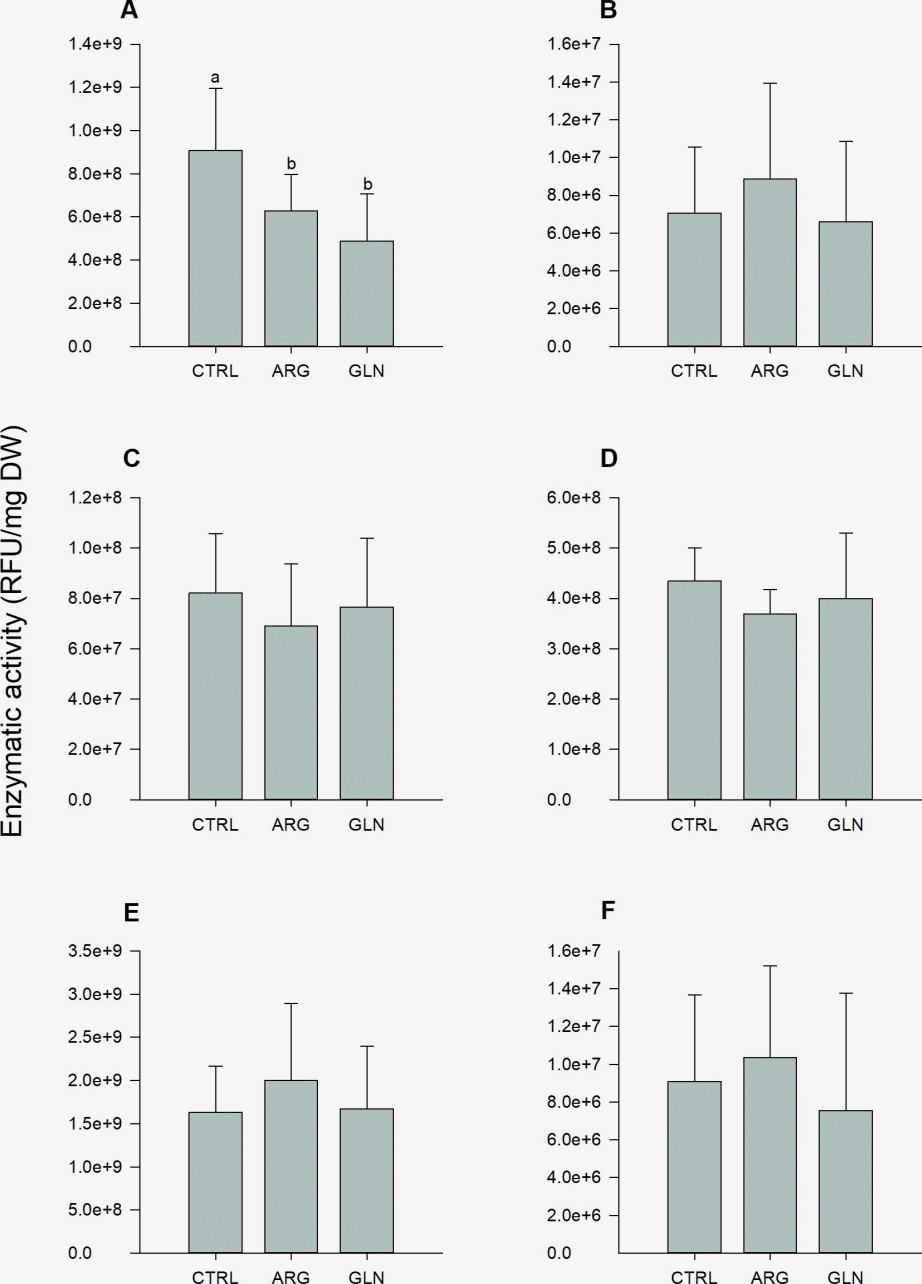
Digestive enzyme activities in zebrafish larvae. Trypsin (A), chymotrypsin (B), aminopeptidase (C), alkaline phosphatase (D), 7C-like lipase (E) and 18C-like lipase (F) activities in zebrafish larvae (22 dpf) from control (CTRL), arginine (ARG) and glutamine (GLN) treatments. Activity values are expressed as RFU/mg larval dry weight. Results are represented as means ± SD (*n* = 6). Different letters (a,b) indicates significant differences between treatments (P<0.05).

The AA supplementation had a significant impact on larvae trypsin activity. ARG and GLN larvae showed the lowest trypsin activity levels when compared with CTRL larvae (6.27E+08, 4.87E+08 and 9.06E+08 RFU/mg DW, respectively) (Fig 3). Chymotrypsin and lipases activities (for both, 7C- and 18C-like lipases) showed similar trends, with a tendency of higher enzymatic activity in ARG larvae. Average values for these enzymes were 7.5E+06, 1.77E+09 and 8.99E+06 RFU/mg DW, chymotrypsin, 7C- and 18C-like lipases, respectively (Fig 3). On the other hand, aminopeptidase and alkaline phosphatase activities showed a trend to decrease in ARG treatment when comparing to remain treatments. Activity average values for these enzymes were 7.59E+07 and 4.01E+08 RFU/mg DW, aminopeptidase and phosphatase alkaline, respectively (Fig 3). PCA demonstrated no clear separation between samples for digestive enzymes activity levels (S2 Fig)

### Gut microbiota composition

Bacterial 16S rRNA V2-V3 regions of larvae polled GITs were analysed in triplicate. HTS analysis yielded a total of 1116534 raw sequences, after removing chimeric sequences a total of 530574 reads were used for downstream analysis. These comprised 115797 sequences were from CTRL larvae, 353730 from ARG larvae and 61047 sequences from GLN larvae. Sequences were clustered into 730 OTUs representing 333 different taxa. Data were deposited in the Zenodo Repository: https://doi.org/10.5281/zenodo.4537443 [31].

**Fig 4:**
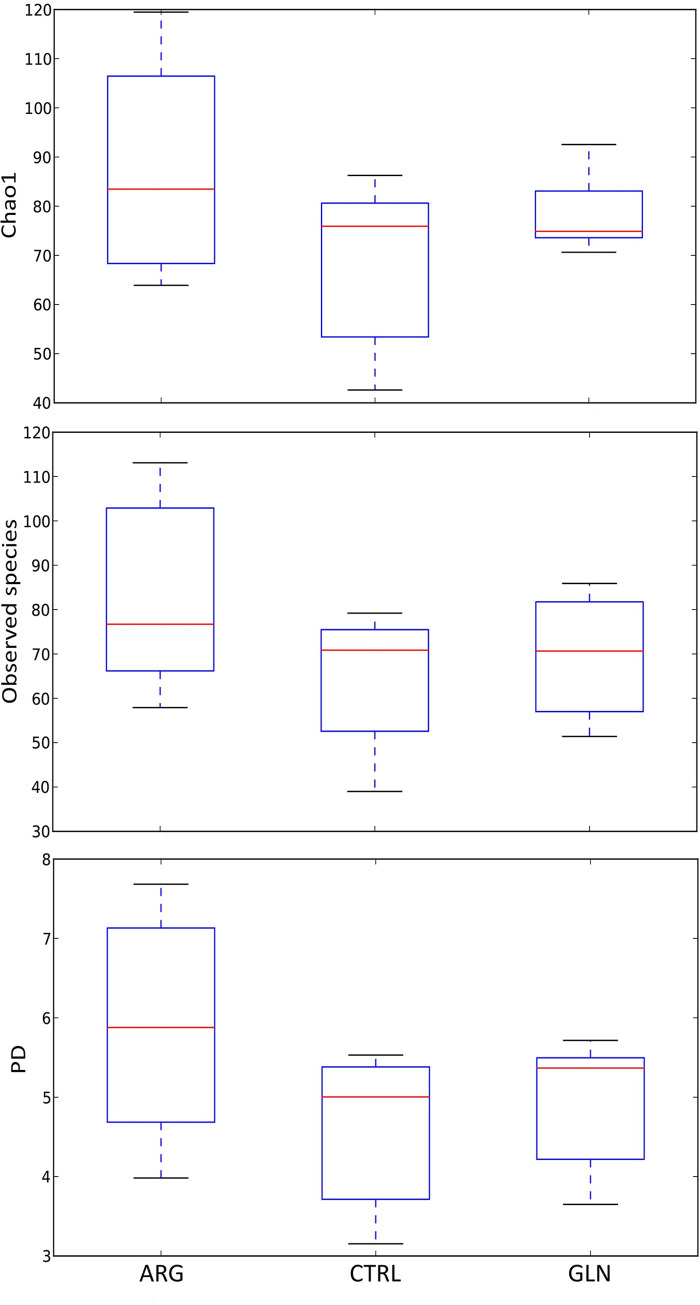
Alpha diversity parameters. Boxplots for Alpha parameters results; Chao1 index, Observed species and phylogenetically diverse (PD) whole tree (operational taxonomic units [OTUs]) of zebrafish larvae GITs from control (CTRL), arginine (ARG) and glutamine (GLN) treatments.

Alpha diversity parameter was not significantly affected (p > 0.05) by the AA supplementation (Fig 4). However, Chao1, the number of observed species and the phylogenetic diversity tended to be higher in ARG larvae.

**Fig 5:**
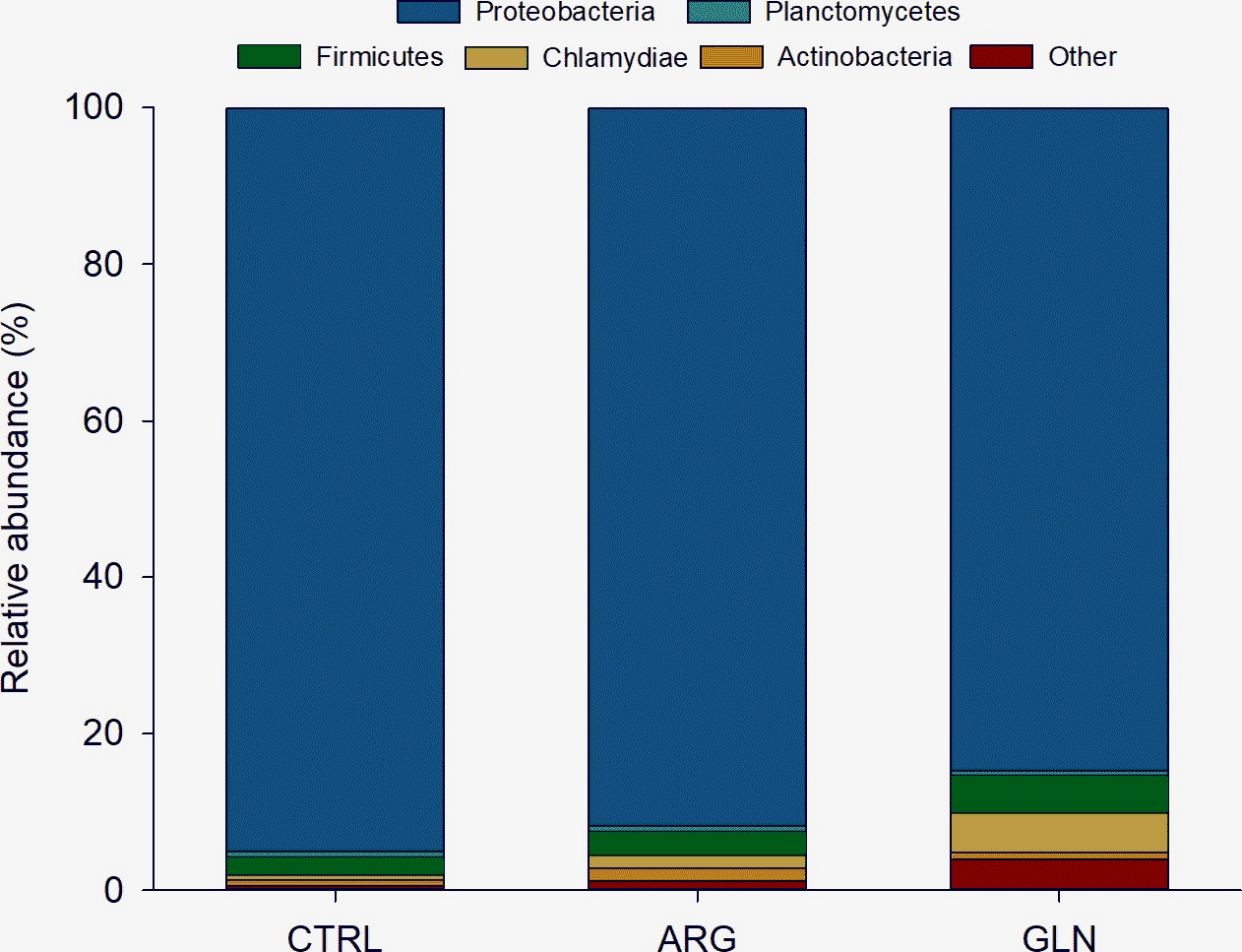
Composition of gut bacterial ecosystem at phylum level. Taxonomic composition of the average (%) phyla level of gut microbiota of zebrafish larvae (22 dpf) from control (CTRL), arginine (ARG) and glutamine (GLN) treatments. The plots represent the most abundant representatives’ phylum (> 1%).

**Fig 6:**
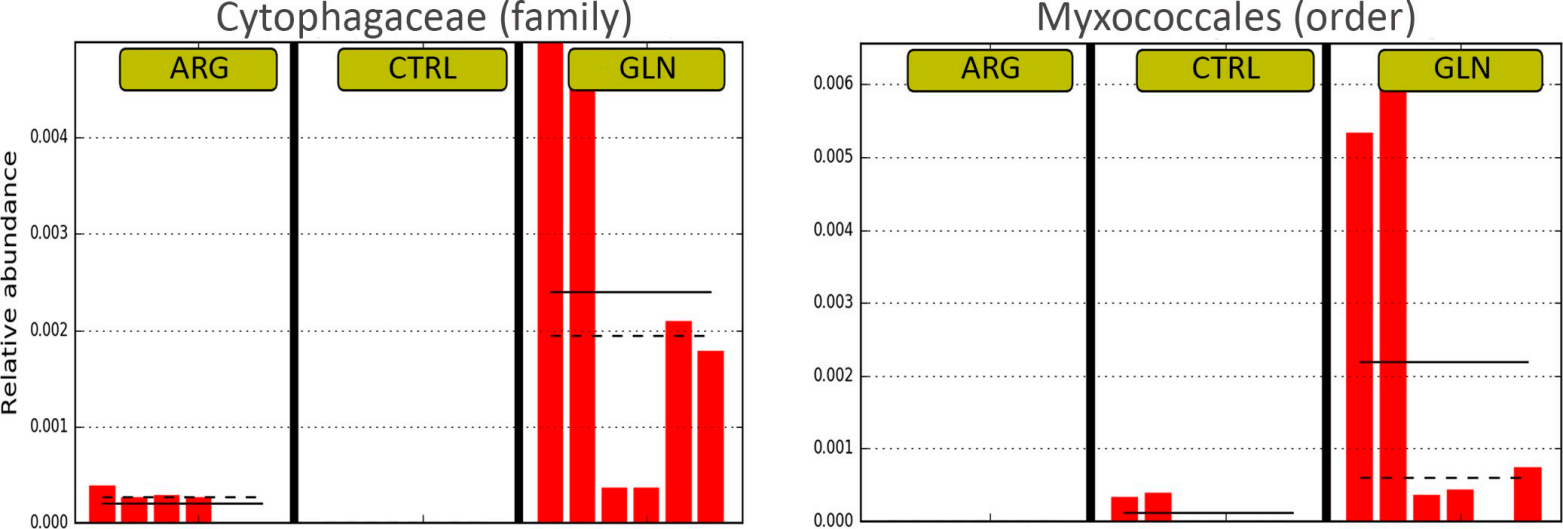
Representation of differences in gut bacterial composition between treatments. Differential features plots for Cytophagaceae (family) on left, and Myxococcales (order) on right showing higher abundance in GLN treatment. Dashed line corresponds to median and full line corresponds to average between samples from the same treatment.

The composition of the gut bacterial ecosystem is shown at phylum level in Fig 5. Proteobacteria comprised more than 84% of the total bacterial proportion in all treatments. The Firmicutes and Chlamydiae tend to be higher in GLN group when compared to CTRL and ARG gut bacterial ecosystem. However, no significant differences were observed in phyla abundance due to supplementation. A significant higher abundance of Cytophagaceae (family) and Myxococcales (order) were observed in GLN larvae (0.23% for both) (Fig 6).

The relative abundance of reads assigned to the genera level (Table 2) was higher for Aeromonas (ranging from 51.2 to 65.6%, GLN and CTRL, respectively) followed by Gemmobacter-Rhodobacter (8.4%), Gemmobacter (3.7%) and Pseudomonas (2.6%).

**Table 2:** Composition of gut bacterial ecosystem at genus level.

Treatment	Aeromonas	Gemmobacter-Rhodobacter	Gemmobacter	Pseudomonas
CTRL	65.6±9.4	8.0±2.1	1.9±1.3	2.7±3.8
ARG	54.5±28.4	10.1±8.6	6.2±8.9	3.3±3.6
GLN	51.2±22.0	7.0±6.5	3.1±2.0	1.8±1.1

Values are means (%) of relative abundance (± SD). Table shows the four most abundant representatives’ genera.

Gut microbiota at genus level of zebrafish larvae (22 dpf) from control (CTRL), arginine (ARG) and glutamine (GLN) treatments.

**Fig 7:**
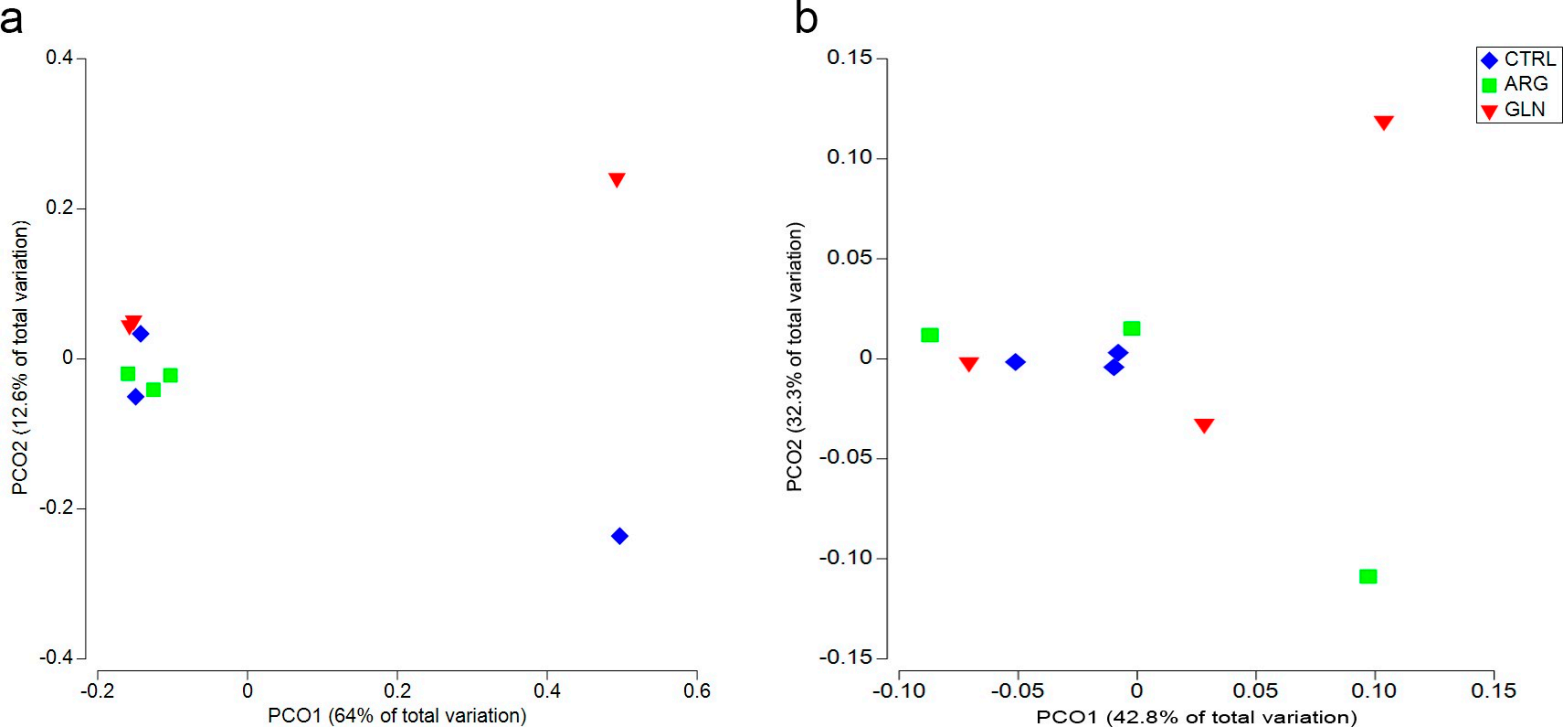
Principal coordinate analysis (PCoA) of unweighted and weighted UniFrac. Principal coordinate analysis (PCoA) of Unweighted (a) and Weighted (b) UniFrac for zebrafish larvae (22dpf) from control (CTRL), arginine (ARG) and glutamine (GLN) treatments.

PERMANOVA analysis did not revealed significant differences between treatments either for weighted or unweighted UniFrac (Table 3). Consequently, principal coordinate analysis (PCoA) plots demonstrate similar results. There was no clear separation between samples in unweighted or weighted UniFrac data (Fig 7A and 7B, respectively), apart from some single samples which appear separate from the main cluster.

**Table 3:** PERMANOVA results of unweight and weighted UniFrac.

		Pairwise test		
	PERMANOVA	CTRL x GLN	CTRL x ARG	GLN x ARG
**Unweighted**				
*P*-value	0.758	0.629	0.901	0.809
Pseudo-*F*/*t*-value	0.68134	0.65316	0.91774	0.96334
**Weighted**				
*P*-value	1.0	0.89	1.0	1.0
Pseudo-*F*/*t*-value	0.42604	0.78175	0.52546	0.65678

PERMANOVA results of unweight and weighted UniFrac between intestinal microbiota composition of zebrafish (*Danio rerio*) larvae from control (CTRL), glutamine (GLN) and arginine (ARG) treatments.

## Discussion

The present study assesses both the effect of an early amino acid supplementation during the embryogenesis of zebrafish as promoter of gut maturation in 22 dpf larvae, as well as the potential of sonophoresis technique for nutritional modulation in fish embryos.

Survival rate at the end of the present study was around 50%, lower than the values commonly obtained for zebrafish larvae rearing (around 80%), however survival was not influenced by the treatments. Since similar values were observed for NO SONO larvae, which were neither supplemented nor submitted to the sonophoresis protocol, results indicate that was not a negative impact of the applied treatments. Similar survival results were obtained by Farias and Certal [32] and Carvalho [33] in zebrafish larvae fed only on inert diet until 30 dpf. The supply of live prey during the first life stages is a common practice in zebrafish rearing, being weaned at juvenile stage (around 30 dpf). In the present study, to assess a positive modulation on digestive capacity due to *in ovo* amino acids supplementation, a challenging feeding protocol was designed based only on inert diet from mouth opening. Martins [34] evaluated the effect of an early weaning on zebrafish, at 10 dpf, on growth, skeletal anomalies, and reproductive performance, obtaining promising results with high quality microdiets and an average survival of 80%. Likewise, Farias and Certal [32] obtained similar survival results in larvae fed on co-feeding or exclusively on live prey. Comparison between results suggest that a short period of live prey should be included in zebrafish larvae feeding protocols to attain higher survival rates.

Larval dry weight at the beginning of exogenous feeding (5 dpf) was similar between treatments, suggesting that amino acids supplementation at embryonic stage did not affect the yolk absorption and amino acids metabolic utilization. The absence of differences in the short-term, changed at later developmental stages. At the end of the experiment (22 dpf), larvae supplemented with glutamine (GLN) were larger than not supplemented larvae (CTRL), revealing a beneficial effect of glutamine on growth performance. ARG larvae reached intermediate growth values, which suggest the potential of also this amino acid as growth promoter, but further studies focused on the optimal arginine concentration need to be addressed to further explore this hypothesis. Growth performance results in the present study are in accordance with the data obtained by Martins [34] in larvae weaned at 10 dpf, confirming that larvae attained expected growth rates although exposed to a challenging feeding protocol. Few studies have previously tested the enrichment of fish eggs with amino acids with the aim of improving larval performance. In a previous study, gilthead seabream (*Sparus aurata*) eggs were enriched with taurine through electroporation with the aim of improving larval performance, however taurine enrichment did not promote growth in 10 dph larvae [12]. Currently, Lopes [35] is the only study assessing *in ovo* incorporation of indispensable amino acids through sonophoresis as growth promoters in fish. In that study methionine was supplemented in gilthead seabream embryos with the objective of increasing the indispensable amino acids pool, that may be a limiting factor for larval growth performance. As expected, growth results were higher in the larvae from methionine supplementation during the first week after hatching. By contrast, the effects of *in ovo* feeding of amino acids have been widely studied in other animals, such as broiler chicken and Indonesian native chicken. *In ovo* glutamine supplementation in broiler chickens had a positive impact on weight gain and feed conversion ratio [36]. In Indonesian native chicken, an *in ovo* injection of glutamine increased hatching rate, decreased the duration of incubation period, and enhanced the weight of newly hatched chickens [37]. Results of the present study revealed that a single *in ovo* stimulus with glutamine can promote growth performance in zebrafish larvae at least until 22 dpf. However, longer trials might be performed to evaluate if this growth performance potential is maintained in the long-term.

Eggs FAAs profile supports the efficiency of sonophoresis technique for *in ovo* nutritional modulation in fish. Higher arginine and glutamine levels were observed in eggs from ARG and GLN treatments, respectively. ARG eggs almost doubled CTRL eggs arginine content, while GLN eggs showed almost a four-fold increase in glutamine content in comparison with CTRL eggs. In Lopes [35], the methionine solution used for sonophoresis was 50-fold more concentrated than methionine levels in gilthead seabream eggs, reaching an incorporation efficiency in the eggs of 33-fold compared to control eggs. Altogether, results indicate that the incorporation efficiency may be different between amino acids [38–40]. Since sonophoresis technique promotes a passive transport through the membrane channels, the compound-incorporation efficiency might be dependent on osmotic regulation. It seems that amino acids supplementation (GLN and ARG treatments) promoted a higher utilization of the indispensable amino acids (IAA). This IAA utilization was visible on the higher growth observed at 22 dpf, at least in the GLN larvae. Moreover, at the end of the experiment (22 dpf), larvae FAA profile was correlated with the profile found at egg stage for each treatment, with higher arginine and glutamine levels in ARG and GLN larvae, respectively. This suggests that fish did not compensate or metabolized the altered FAA levels along the larval development. That is also noticeable by the maintenance of the IAA:DAA ratios between egg and larvae stage. It has been described that IAA in fish are preferentially destinated to growth purposes while DAA are preferentially used as an energy substrate [41]. Thus, higher DAA levels might have served as energy substrate to support the higher growth performance observed for GLN larvae at the end of the experiment. On the other hand, although ARG larvae presented higher IAA levels, the lack of energy fuel to support higher growth rates might have been limiting for this treatment to achieve better growth performance. Previous studies dietary used arginine supplementation as a mechanism to boost the immune system in mammals, but also in fish [42]. In juvenile channel catfish (*Ictalurus punctatus*) dietary arginine enhanced some immune system biomarkers, such as haemoglobin, haematocrit, and circulating erythrocytes [43]. Similarly, dietary arginine supplementation led to positive changes to several components of the innate immune system of red drum [7]. In the present study the larval immune response was not evaluated, however, the previously described results suggest that ARG larvae might present a more robust immune system, which might justify the smaller metabolic investment in growth compared to GLN larvae. Further experiments including a stress event are needed to assess if an embryonic stimulus with arginine might enhance the immune response in zebrafish larvae. Altogether, results showed that an early modulation of the embryo FAAs profile may have an impact on larval growth as well as an imprint on the regulation of larval AA metabolism.

Overall, FAAs levels tended to decrease with larval development. This is an expected pattern observed also in other species as starry flounder (*Platichthys stellatus*) [44], in which the decrease in FAAs was linked to an increase in the protein amino acids (PAAs), indicating that morphogenesis converts soluble matter (FAAs) into insoluble matter (PAAs). Similar results were described for catfish (*Clarias gariepinus*) in a study analysing ontogenetic changes in the total AA profile. This study concluded that the changes observed in the larval AA profile during ontogeny probably reflected a change in the proportions of the various proteins being synthesized during larval growth and/or the allometric growth of the larvae, as different organs and tissues develop at varying rates and times [45].

Protein and amino acids are an essential component in the maintenance of general health and well-being [46]. In fish, many studies have shown the therapeutic effect of amino acids for growth as well as to maintain metabolism [47,48]. The exocrine pancreas synthesizes and secretes digestive enzymes and requires optimal nutrition for enzyme synthesis. In addition, the supply of a high-AA diet to juvenile rats promoted pancreatic growth and proteases secretion [49]. In the present study digestive enzymes activity patterns were similar between larvae regardless of the treatment, except for trypsin activity, which was significantly lower in the *in ovo* amino acid-supplemented larvae. Some studies have shown that dietary AAs can increase pancreatic trypsin levels in juvenile rats [49,50]. Supplementation of glutamine in newly hatched broiler chicken diets led to an improved average body weights and to an increase in digestive enzyme activities (trypsin, lipase, and amylase) [51].

Likewise, studies have shown that arginine play critical roles in promoting gut functions and digestive capacity [52]. Specifically, for fish species, few studies assessed the effect of glutamine or arginine on the early modulation of the digestive function, however several studies have tested both amino acids as dietary supplements for juvenile fish. Half-smooth tongue sole (*Cynoglossus semilaevis*) larvae supplemented with dietary glutamine showed higher trypsin, amylase and brush-border enzymes activities when compared to untreated larvae, improving digestive functionality [53]. In Jian carp (*Cyprinus carpio* var. Jian), dietary arginine increased intestine and hepatopancreas protein content, as well as trypsin, chymotrypsin, lipase, and alkaline phosphatase activities [54]. In juvenile hybrid sturgeon (*Acipenser schrenckii*×*Huso dauricus*) similar results were observed, with increased protease, lipase, and amylase activity levels in glutamine supplemented fish [55]. Despite this, the opposite effect on trypsin activity was observed in this study after an embryonic supplementation with either glutamine or arginine. Lower tryptic activity was recorded in GLN and ARG larvae compared to CTRL larvae, that probably affected rates of protein digestion and amino acid absorption. However, GLN larvae presented a higher weight at the end of the trial. In juvenile Jian carp, dietary glutamine supplementation improved weight gain, feed intake and digestive enzyme activities [8]. Although larvae feed intake was not measured in the present study, an increase in feed ingestion together with a more efficient metabolism might explain the higher growth performance in GLN larvae.

Microbial colonisation of fish larvae originates from the eggs, the surrounding water and the first feeding. Several studies have shown that diet is influential in shaping the gut microbial community and from first feeding substantial diversification occurs [56,57]. Microorganisms within the GIT are involved in the regulation of appetite/ingestion, digestion, and metabolism. Since metabolites from the gut microbiota can modify the secretory activity of enterocytes, the production of gut peptides that modulate enzyme secretion can be affected, regulating absorptive capacity and nutrient uptake and storage [58]. That is why there has been a growing interest in the manipulation of fish gut microbiota to improve welfare and nutrition. The principal methods of gut microbial manipulation have included the alteration of dietary proteins and lipids, as well as the addition of probiotics and prebiotics in the diet [57]. However, this is the first study assessing the modulation of fish microbiota from the embryonic stage through early nutritional programming. It has been described for both, glutamine and arginine, their potential to effectively act on the microbiota in the intestine to shift the bacterial diversity and regulate the homeostasis and equilibrium of AA [59,60]. In the present study, embryonic amino acid supplementation did not lead to differences in larval bacterial diversity. ARG larvae showed a trend to higher values of alpha diversity parameters. Similar results were observed in mice, where arginine dietary supplementation did not affect the total number of bacteria in the colonic faeces, but the diversity of the intestinal microbiota was significantly enhanced in mice that were given the high-arginine supplementation diet compared to the two other diets (low-arginine supplementation and control) [60]. It is important to highlight the high dispersion recorded in the present results. A high variation in the composition of the microbiota among individual larvae within one tank has been described also for Atlantic cod (*Gadus morhua*), comparable to the variation between rearing facilities with different holding regime [61]. Further studies are needed to verify whether this individual variation is characteristic for fish larvae, but especially, to what extent this broad variation in microbiota of larvae has biological implications for the host. In that sense, Vadstein [62] suggested that the functions needed in the larval bacterial ecosystem might be promoted by different species inventory, and not a specific species composition. The zebrafish intestinal habitat has been described to select for specific bacterial taxa despite radical differences in host provenance and domestication status [63]. The bacterial groups found in the present study are in line with the most representative groups described previously for zebrafish microbiota, dominated by Proteobacteria, Firmicutes and Actinobacteria at phyla level, and genera Aeromonas [63,64]. Those studies also included Fusobacteria, which are typically obligate anaerobes, between the dominant phyla in zebrafish microbiota, however, it seems to be a major group in adult fish. The explanation to this colonization pattern through fish ontogeny might be that as the digestive tract and associated bacterial communities mature, the environment becomes anoxic, opening a niche for obligate anaerobes [65]. GLN larvae revealed higher levels of Cytophagaceae. Many members of the family Cytophagaceae are shown to digest macromolecules such as polysaccharides or proteins [66], which might have contributed to a higher nutrient digestion, probably compensating the lower proteolytic activity in GLN larvae, thus, supporting a higher growth. Nonetheless, early amino acid supplementation did not show a marked effect on zebrafish GIT microbiota profile, suggesting that further studies are needed to find out the mechanisms of early microbiota modulation in fish larvae.

In conclusion, the results of the present work indicate that some amino acids may act as promoters of early intestinal maturation, improved digestive capacity and growth performance in fish larvae. We also identified a “developmental window” in which nutritional programming has short-term effects on digestive functionality. Glutamine supplementation at zebrafish embryonic stage was able to enhance performance at later developmental stage even with lower trypsin activity, suggesting a higher nutrient digestion capacity, due to a slightly modulation of GIT microbiota. Higher arginine supplementation levels should be tested as strategy to enhance growth at later developmental stages. Overall, the present study supports sustainable self-sufficiency in fish aquaculture by integrating dietary strategies and relevant data obtained from new tools. The perspective of applying this novel concept to aquaculture industry provides numerous advantages, since digestive capacity is considered key to fish resilience and quality. However, further, and longer studies with different bioactive compounds and concentrations are needed to understand the mechanisms modulating early microbial colonization with the objective of improving fish robustness and resilience in the long-term.

## Supporting information

S1 Fig FAA profile PCA analysis. Principal Component Analysis (PCA) of free amino acids (FAA) profile of zebrafish embryo 1h after sonophoresis (a) and 22 dpf larvae (b) from CTRL, ARG and GLN treatments.
(TIF)

S2 Fig Digestive enzyme activities PCA analysis. Principal Component Analysis (PCA) of digestive enzymes activity levels in 22 dpf zebrafish larvae from CTRL, ARG and GLN treatments.
(TIF)
