# Supplementary figures and images for: Egg nutritional modulation with amino acids improved performance in zebrafish larvae
Source: PLoS One. 2021 Apr 9;16(4):e0248356. doi: 10.1371/journal.pone.0248356 (PMC8034726; doi:10.1371/journal.pone.0248356)

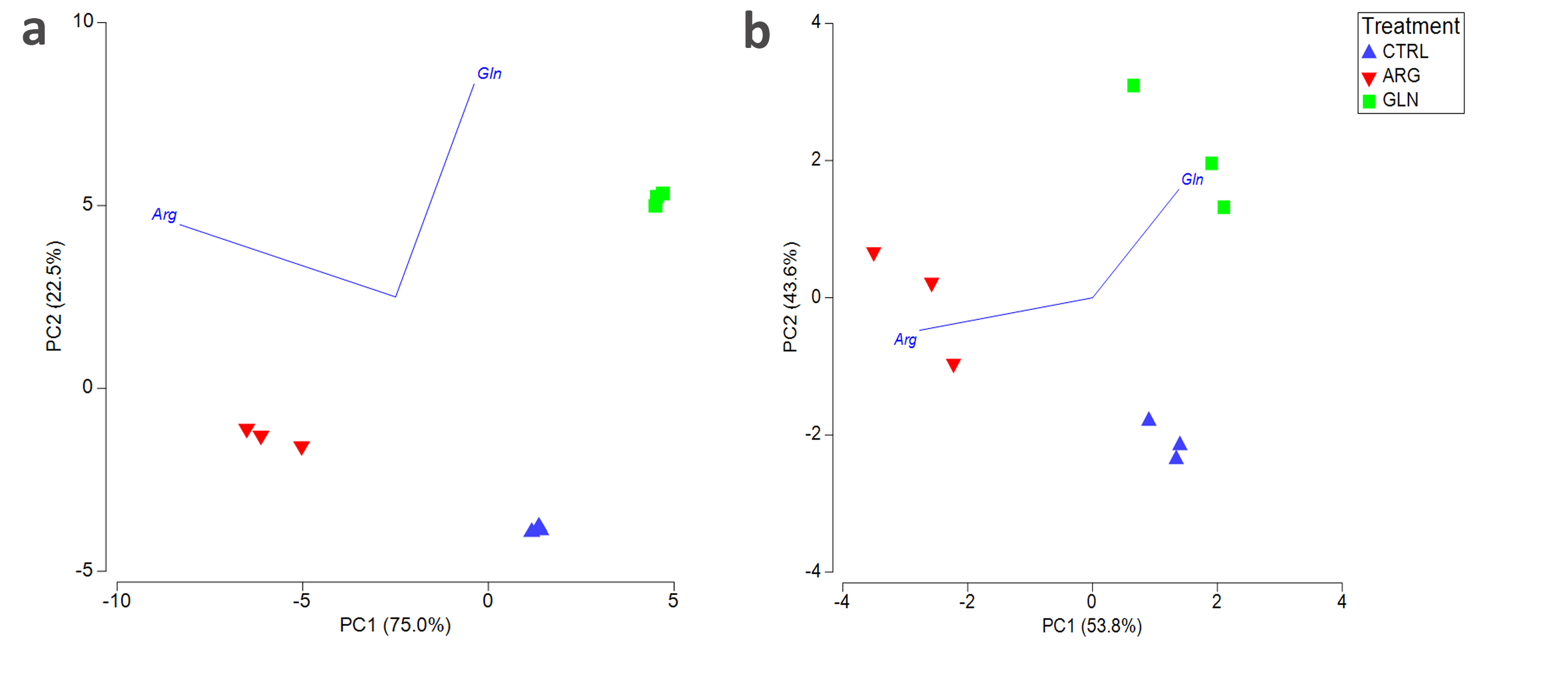

Supplement: S1 Fig — Principal Component Analysis (PCA) of free amino acids (FAA) profile of zebrafish embryo 1h after sonophoresis (a) and 22 dpf larvae (b) from CTRL, ARG and GLN treatments. (TIF) [file pone.0248356.s001.tif]

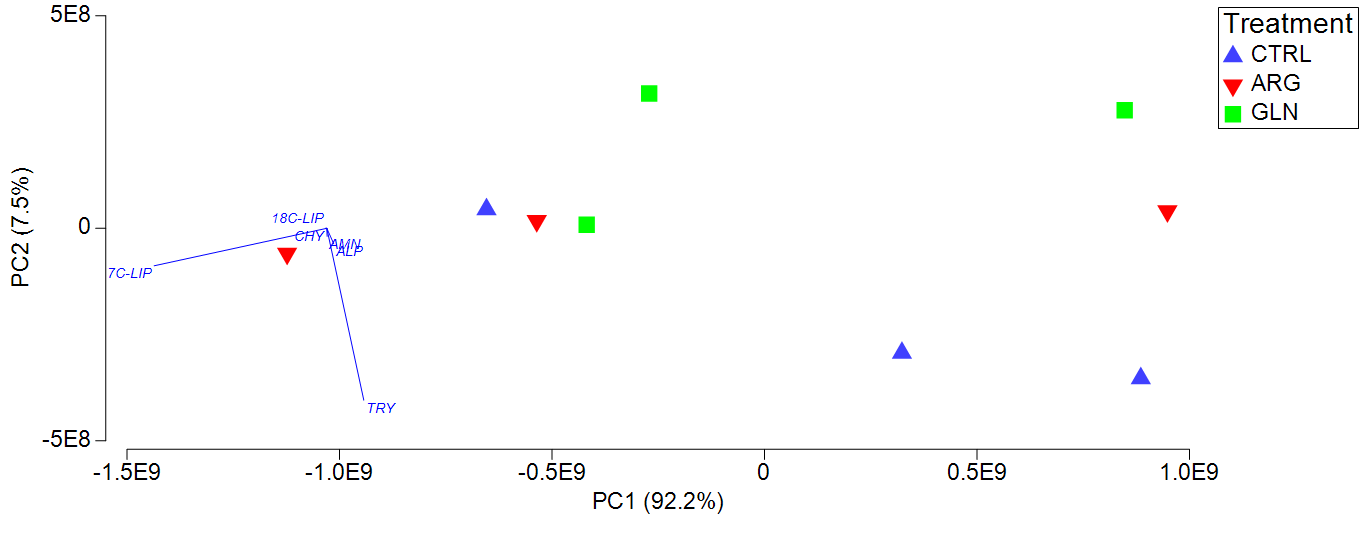

Supplement: S2 Fig — Principal Component Analysis (PCA) of digestive enzymes activity levels in 22 dpf zebrafish larvae from CTRL, ARG and GLN treatments. (TIF) [file pone.0248356.s002.tif]
